# Effects of Periodontal‐Specific Exosomes and rhBMP2 on Osteogenic Behaviour and Differentiation of BMSCs

**DOI:** 10.1111/jcmm.71039

**Published:** 2026-01-28

**Authors:** Paras Ahmad, Danyal A. Siddiqui, Jared Bianchi‐Smak, Nima Farshidfar, Nathan Estrin, Richard J. Miron, Georgios A. Kotsakis

**Affiliations:** ^1^ Department of Oral Biology, Rutgers School of Dental Medicine The State University of New Jersey Newark New Jersey USA; ^2^ Clinical Research Center, Rutgers School of Dental Medicine The State University of New Jersey Newark New Jersey USA; ^3^ Department of Research Advanced PRF Education Jupiter Florida USA; ^4^ Department of Biological Sciences Rutgers University Newark New Jersey USA; ^5^ Department of Periodontology University of Bern Bern Switzerland; ^6^ Department of Periodontology Lake Erie College of Osteopathic Medicine School of Dental Medicine Bradenton Florida USA

**Keywords:** amniotic fluid, bone marrow cells, bone morphogenetic protein 2, exosomes, mesenchymal stem cells

## Abstract

Growth factors, including recombinant human bone morphogenetic protein‐2 (rhBMP2), have been clinically utilised for large bone augmentation with good outcomes. Nevertheless, long‐term healing, swelling, safety concerns, and high cost limit their use. Exosomes, nanoscale extracellular vesicles, have emerged as promising regenerative alternatives. This study assessed the osteogenic potential of periodontal‐specific exosomes (Px) on bone marrow mesenchymal stem cells (BMSCs) compared to rhBMP2. Px were morphologically characterised by TEM and quantified via BCA assay. BMSCs were treated with Px at 1:10, 1:50, and 1:100 dilutions (100, 20, and 10 μg/mL) and compared to rhBMP2 (100 ng/mL). Px uptake was evaluated using PKH26 labeling. Functional assays included viability, migration, alkaline phosphatase (ALP) activity, alizarin red (ARS) mineralization, collagen, osteocalcin secretion, and RT‐PCR analysis of osteogenic genes. Px exhibited spheroidal to cup‐shaped morphology and internalisation in BMSCs up to 18 days. Compared to rhBMP2, Px promoted viability (1.14‐fold), migration (1.78‐fold) up to 1.14 and 1.78‐fold, ALP (1.48‐, 4.11‐fold), ARS (1.43‐, 14.71‐fold), collagen (1.40‐, 3.58‐fold), and osteocalcin (1.86‐, 5.2‐fold). Gene expression demonstrated significant upregulation of *ALP* (1.73‐fold), *RUNX2* (1.70‐fold), *OCN* (1.36‐fold), and *OPN* (1.35‐fold). Overall, Px significantly enhanced BMSC osteogenesis compared to rhBMP2, highlighting their potential as a cell‐free nanotherapeutic in bone tissue engineering.

## 1 Introduction

Bone regeneration represents a significant challenge in both craniofacial and orthopaedic reconstruction, especially when complex or large defects are present [1]. Conventional treatment approaches, such as synthetic biomaterials, allografts, and autografts, are extensively utilised but are restricted by significant disadvantages, including suboptimal osseointegration within host tissue, limited graft availability, potential immune rejection, and donor‐site morbidity [2, 3]. Resultantly, there is an increasing need for innovative, biologically driven approaches that can effectively facilitate bone healing and regeneration.

Among these strategies, mesenchymal stem cells (MSCs) have emerged as fundamental components of regenerative strategies owing to their immunomodulatory characteristics, multipotency, and self‐renewal capabilities [4]. Bone marrow‐derived mesenchymal stem cells (BMSCs) in particular have been extensively investigated for their capability to differentiate into osteoblasts and contribute directly to osteogenesis [5]. To promote their osteogenic effect, growth factors, including bone morphogenetic protein‐2 (BMP2), have been extensively used in experimental and clinical settings [6]. While recombinant human (rhBMP2) is a potent osteoinductive agent, its clinical application is limited by numerous important limitations, including post‐surgical complications, ectopic bone formation, inflammatory swelling, brief biological half‐life, and high cost [7, 8]. These limitations have prompted the search for alternative biologic approaches that can offer robust osteogenic stimulation with enhanced biological control and safety.

Over the recent years, growing evidence indicates that numerous therapeutic advantages conventionally associated with MSCs are regulated not by the cells themselves, but by the bioactive molecules they release, especially extracellular vesicles (EVs) referred to as exosomes [9]. Exosomes are nanosized vesicles (nearly 30–150 nm in diameter) originating from multivesicular bodies and released into the extracellular space, where they serve as primary regulators of intercellular communication [10, 11]. They encapsulate diverse molecular cargo, including nucleic acids (DNA, messenger RNAs, and microRNAs), transcription factors, lipids, and proteins, which can regulate the behaviour of target cells [12, 13]. Through endocytic uptake, membrane binding, or receptor fusion, exosomes modulate necessary cellular processes, including proliferation, migration, differentiation, angiogenesis, and inflammation [14, 15, 16]. Importantly, exosome‐based therapies are considered “cell‐free”, hence reducing the risks associated with live cell transplantation while retaining much of the regenerative potency of their parent cells [9].

Exosomes derived from MSCs (MSC‐Exos) have emerged as central effectors of tissue regeneration across multiple organ systems [17, 18]. A large body of evidence has shown that MSC‐Exos enhance angiogenesis, promote cell viability, regulate immune responses, and activate tissue‐targeted progenitor cell differentiation via the delivery of regulator proteins, growth factors, and miRNAs [19, 20]. In bone tissue engineering, MSC‐Exos have been demonstrated to stimulate osteogenic signalling cascades, including phosphoinositide 3‐kinase (PI3K)/protein kinase B (Akt), mitogen‐activated protein kinase (MAPK), and canonical Wnt signalling (Wnt/β‐catenin) pathway, resulting in the upregulation of osteopontin (OPN), osteocalcin (OCN), alkaline phosphatase (ALP), and runt‐related transcription factor 2 (RUNX2), together with collagen formation and matrix mineralization [21, 22]. Notably, MSC‐Exos can reproduce several of the regenerative effects of their donor cells while bypassing vital drawbacks of cell therapy, such as poor cell viability after transplantation, tumorigenicity, and immune rejection [23]. These benefits position exosomes as a clinically stable and biologically sophisticated approach for bone regeneration.

The ability of periodontal‐specific exosomes (Px) to enhance osteogenic differentiation of BMSCs has yet to be rigorously assessed. Moreover, their comparative effectiveness against traditional osteoinductive agents such as BMP2 remains uncertain. To address these gaps, the present study aimed to explore the osteogenic potential of Px on BMSCs and to compare its efficacy against the conventional osteoinductive agent, i.e., rhBMP2. We hypothesised that Px would enhance osteogenic differentiation and mineralization of BMSCs to a greater extent than rhBMP2. By systematically examining the bioactivity of Px across various aspects of osteogenesis, this study endeavors to lay the groundwork for the advancement of exosome‐mediated therapies for bone regeneration.

## 2 Material and Methods

Table S1 shows the list of materials utilised.

### 2.1 Cell Culture

BMSCs were expanded in MSC expansion medium under standard culture conditions at 37°C in a humidified atmosphere containing 5% CO_2_ and 95% air. Once the cells reached approximately 80% confluence, sub‐passaging was performed by enzymatic dissociation using 0.25% (w/v) trypsin–EDTA solution. BMSCs at passages 4 to 6 were used for all experiments to ensure consistency.

### 2.2 Morphological Characterisation of Px

Px were obtained from NeoBiosis (Gainesville, FL, USA) as proprietary exosome preparations manufactured under Good Manufacturing Practices (GMP) standards. Px were morphologically characterised using transmission electron microscopy (TEM), following established protocols [24]. Briefly, 3 μL of Px suspension in saline was carefully applied to a glow‐discharged, carbon‐coated copper grid and allowed to adsorb for 3 min. Excess liquid was gently removed using filter paper. The grid was then negatively stained with 5 μL of 0.75% (w/v) uranyl formate for 1 min. Following staining, the grid was air‐dried overnight at room temperature. The morphological assessment of Px was performed using an electron microscope operated at an accelerating voltage of 80 kV. Images were captured with a GATAN OneView 4k × 4k CMOS camera.

### 2.3 Quantification of Total Protein Concentration in Px

The total protein concentration of Px was determined using the bicinchoninic acid (BCA) assay, following the manufacturer's instructions. A standard curve was generated using serial dilutions of bovine serum albumin (BSA) in distilled water at concentrations of 2000, 1500, 1000, 750, 500, 250, 125, 25, and 0 μg/mL. In a 96‐well microplate, 25 μL of each BSA standard or Px suspension (diluted in normal saline) was combined with 200 μL of BCA working reagent (prepared by mixing Reagent A and Reagent B in a 50:1 ratio). The plate was incubated at 37°C for 30 min, and absorbance was subsequently measured at 562 nm using a multimode microplate reader. Protein concentrations of Px samples were calculated based on the BSA standard curve [25].

### 2.4 Px Labeling and Cellular Uptake

To investigate the internalisation of Px by BMSCs, Px were fluorescently labelled with PKH26 [26] using the PKH26 Red Fluorescent Cell Linker Midi Kit, following the manufacturer's instructions. Briefly, 20 μg of Px were resuspended in 250 μL of Diluent C (Part A), while 1 μL of the PKH26 dye was diluted in 250 μL of Diluent C (Part B). The two solutions were then combined and incubated for 5 min at room temperature to permit labeling. The reaction was quenched by adding 500 μL of 1% BSA for 1 min. The labelled Px were washed with phosphate‐buffered saline (PBS) and purified by ultracentrifugation at 100,000× *g* for 70 min at 4°C [19]. BMSCs were seeded in a glass‐bottom cell culture plate and incubated with PKH26‐labelled Px at a concentration of 20 μg/mL for 24 h at 37°C under standard culture conditions. Following incubation, the cells were washed three times with PBS and fixed with 4% formaldehyde (FA). F‐actin was visualised using ActinGreen 488 ReadyProbes reagent, and nuclei were counter‐stained with DAPI using NucBlue Fixed Cell Stain ReadyProbes reagent solution. Fluorescence imaging was performed using a Leica DM i8 inverted fluorescence microscope to evaluate the cellular uptake and distribution of Px.

### 2.5 Live/Dead Staining

The viability of BMSCs following treatment with Px (1:10, 1:50, and 1:100) and rhBMP2 (100 ng/mL) was evaluated using a live‐dead staining assay. BMSCs were seeded in a 12‐well plate at a density of 50,000 cells/well in 1 mL of basal culture medium and treated with Px and rhBMP2 for 24 h. After treatment, the cells were washed with PBS and incubated with 2 μmol/L Calcein‐AM to stain live cells and 4 μmol/L propidium iodide (PI) to stain dead cells. The staining was conducted by adding the reagents directly to the wells, followed by incubation for 15 min at 37°C. Fluorescent images were captured. Live and dead cells were quantified by counting the number of Calcein‐AM‐positive (green) and PI‐positive (red) cells in 4 randomly selected microscopic fields per well.

### 2.6 Transwell Migration Assay

Cell migration was assessed using a transwell migration assay with polycarbonate membrane inserts containing 8‐μm pore filters. Briefly, 50,000 BMSCs were seeded into the upper chambers of transwell inserts in serum‐free medium. The lower chambers were filled with 1 mL of culture medium supplemented with Px (1:10, 1:50, and 1:100) and rhBMP2 (100 ng/mL). After 24 h of incubation at 37°C, the inserts were gently washed three times with PBS. Non‐migrated cells on the upper surface of the membrane were carefully removed using sterile cotton swabs. The membranes were then fixed with 4% FA and stained with 0.5% (w/v) crystal violet solution. Migrated cells on the lower surface of the membrane were imaged using an inverted light microscope. The number of migrated cells was quantified by counting cells in 4 randomly selected microscopic fields per well.

### 2.7 Scratch Wound Assay

For the scratch assay, BMSCs were seeded at a density of 50,000 cells per well in a 12‐well plate and cultured for 24 h to allow cell adhesion and reach around 80% confluency. To minimise cell proliferation during the assay, the culture medium was replaced with low‐serum medium containing 0.5% fetal bovine serum (FBS). A linear scratch (“wound”) was then created across the cell monolayer using a sterile 200 μL pipette tip. Detached cells were gently removed by washing with PBS, and images of the initial wound area were captured immediately. Subsequently, cells were treated with culture medium containing rhBMP2 (100 ng/mL) and Px (1:10, 1:50, and 1:100) at the indicated concentrations. Wound closure was monitored by capturing images at 0, 12, and 24 h post‐scratch using an inverted light microscope. The rate of cell migration into the scratch area was quantified using ImageJ software by measuring the number of cells that migrated into the wound area [27]. All experiments were performed in duplicates, and three randomly selected microscopic fields per well were analysed for quantification.

### 2.8 Osteogenic Differentiation of BMSCs

To induce osteogenic differentiation, BMSCs were cultured until they reached approximately 80% confluency. The basal culture medium was then replaced with osteogenic induction medium prepared using the StemPro Osteogenesis Differentiation Kit, according to the manufacturer's instructions. The differentiation medium was replenished every 3 days.

### 2.9 Collagen Matrix Deposition Assay

To assess extracellular matrix deposition, BMSCs were seeded at a density of 50,000 cells per well and cultured in osteogenic differentiation medium for 14 days in the presence of Px (1:10, 1:50, and 1:100) and rhBMP2 (100 ng/mL). Total fibrillar collagen content was quantified using the Sirius Red/Fast Green Collagen Staining Kit, following the manufacturer's instructions. At the end of the differentiation period, the osteogenic medium was aspirated, and the cells were washed twice with PBS. The cells were then fixed with 500 μL of Kahle's fixative per well for 10 min at room temperature. After fixation, the cells were washed twice with PBS, and 300 μL of Sirius Red/Fast Green dye solution was added to each well to fully cover the fixed cell layers. The plate was incubated for 30 min at room temperature. Following incubation, the dye solution was carefully aspirated, and the wells were rinsed repeatedly with 500 μL of distilled water until the water solution was clear. The stained cell layers were imaged using an inverted light microscope to visualise collagen deposition. For quantitative analysis, 500 μL of dye extraction buffer was added to each well and mixed gently by pipetting until the dye was completely eluted. The extracted dye solutions were transferred to a 96‐well plate, and the absorbance was measured at 540 and 605 nm using a microplate spectrophotometer. The concentrations of total collagen and non‐collagenous proteins were calculated using the following equations [28]:

$$\text{Collagen}\;\left(\mathrm{\mu g}/\text{section}\right)=\left(\mathrm{OD}\;540\;\text{value}-\left[\mathrm{OD}\;605\;\text{value}\times 0.291\right]\right)/0.0378$$

$$\text{Non−collagenous proteins}\;\left(\mathrm{\mu g}/\text{section}\right)=\mathrm{OD}\;605\;\text{value}/0.00204$$

### 2.10 Alkaline Phosphatase Assay

#### 2.10.1 Qualitative Analysis

Qualitative assessment of ALP activity was conducted using an ALP staining kit following the manufacturer's recommendations. BMSCs were seeded at 50,000 cells per well and cultured in osteogenic differentiation medium for 14 days in the presence of Px (1:10, 1:50, and 1:100) and rhBMP2 (100 ng/mL). At the end of the incubation period, the medium was gently aspirated, and the cells were washed twice with 1 mL of 1× PBS containing 0.05% Tween‐20 (PBST). The cells were then fixed with 400 μL of the provided fixing solution per well for 2 min at room temperature. After fixation, the wells were washed twice with 1 mL of 1× PBST to remove residual fixative. Next, 400 μL of ALP staining solution was added to each well, and the plates were incubated at room temperature for 30 min. The staining solution was then aspirated, and the wells were washed three times with PBS to remove excess dye. The stained cell layers were imaged using an inverted light microscope.

#### 2.10.2 Quantitative Analysis

Quantitative measurement of extracellular ALP activity was performed on day 7 using a colorimetric ALP assay kit, following the manufacturer's instructions. This kinetic assay is based on the enzymatic hydrolysis of p‐Nitrophenyl phosphate (pNPP) by ALP, yielding p‐nitrophenol and phosphate, which produces a yellow colour measurable at 405 nm. Briefly, the osteogenic medium was aspirated, and the cells were washed twice with PBS. The cells were then lysed with 500 μL of 0.2% Triton X‐100 in distilled water for 20 min at room temperature. Following lysis, 50 μL of each cell lysate was transferred into wells of a clear, flat‐bottom 96‐well microplate. For assay controls, 200 μL of distilled water (blank) and 200 μL of calibrator were added to separate wells. Subsequently, 150 μL of the working reagent was added to each well, bringing the final reaction volume to 200 μL per well. The plate was gently tapped to mix, and absorbance at 405 nm was measured immediately (*T* = 0 min) and again after 4 min (*T* = 4 min) using a microplate spectrophotometer. ALP activity was calculated using the following formula [29]:

$$\begin{array}{c} \mathrm{ALP}\;\left(\mathrm{IU}/L\;\text{or μmol}/\left(L\;\min\right)\right)=\left({\mathrm{OD}}_{T4}—{\mathrm{OD}}_{T0}\right)\\ \times R_{\mathrm{xn}}\mathrm{Vol}\times 35.3/\left({\mathrm{OD}}_{\mathrm{Cal}}—{\mathrm{OD}}_{\text{Blank}}\right)\times \text{SmplVolxT} \end{array}$$

where OD_T4_ = OD value at 405 of sample at 4 min; OD_T0_ = OD value at 405 of sample at 0 min; OD_Cal_ = OD value at 405 of calibrator; OD_Blank_ = OD value at 405 of blank; RxnVol = Final reaction volume (200 μL); T = Reaction time; SmplVol = The amount of sample used in the reaction.

### 2.11 Alizarin Red Staining

Alizarin Red staining (ARS) was performed to assess calcium deposition following 14 days of osteogenic differentiation of BMSCs treated with Px (1:10, 1:50, and 1:100) and rhBMP2 (100 ng/mL). After aspirating the culture medium, the cells were washed twice with PBS and fixed with 4% PFA for 15 min at room temperature. The fixed cells were then stained with 2% ARS solution for 30 min in the dark. Following staining, the wells were thoroughly rinsed with PBS, and the stained cell layers were imaged using an inverted light microscope. For semi‐quantitative analysis, images were processed using ImageJ software [27]. Grayscale images were first inverted, and background subtraction was performed using the rolling ball algorithm with a radius of 12.5 pixels [30]. Representative regions within each well were selected, and the mean grey values were measured after blank subtraction. The results were expressed as arbitrary intensity units, reflecting the relative extent of calcium mineralization.

### 2.12 Enzyme‐Linked Immunosorbent Assay for Osteocalcin

The secretion of OCN in culture supernatants was quantified using a commercially available enzyme‐linked immunosorbent assay (ELISA) kit, following the manufacturer's recommendations. BMSCs were cultured under osteogenic differentiation conditions in a 12‐well plate, treated with Px (1:10, 1:50, and 1:100) and rhBMP2 (100 ng/mL). Supernatants were collected at day 14 of culture for OCN quantification. Briefly, 25 μL of each sample was added to the respective wells of the ELISA plate, followed by the addition of 100 μL of the anti‐OCN horseradish peroxidase conjugate to each well. The plate was then incubated for 2 h at room temperature. After incubation, the wells were thoroughly aspirated, and each well was washed three times to remove unbound material. Subsequently, 100 μL of chromogen solution was added to each well, and the plate was incubated in the dark for 30 min at room temperature. The reaction was terminated by adding 100 μL of stop solution to each well. The optical density of each well was measured at 450 nm using a microplate spectrophotometer. OCN concentrations were calculated based on the standard curve generated according to the kit's instructions [31].

### 2.13 Gene Expression Analysis

Quantitative real‐time polymerase chain reaction (qRT‐PCR) was employed to evaluate the impact of Px (1:10, 1:50, and 1:100) and rhBMP2 (100 ng/mL) on the expression of osteogenesis‐related genes in BMSCs. Cells were cultured in a 12‐well plate with osteogenic differentiation medium supplemented with rhBMP2 and Px. After 7 and 14 days of induction, total RNA was extracted using the Quick‐RNA Miniprep Kit, following the manufacturer's instructions. RNA concentration and purity were determined using the Qubit RNA High Sensitivity Broad Range Assay kit and measured with a NanoDrop 2000 UV–Vis Spectrophotometer. For each sample, 1 μg of total RNA was reverse transcribed into complementary DNA (cDNA) using the iScript cDNA Synthesis Kit in a final reaction volume of 100 μL. Gene expression was analysed for a panel of osteogenesis‐related markers, including: *GAPDH* (housekeeping gene), *RUNX2*, *ALP*, *OCN*, and *OPN*. The sequences of all primers used in this study are provided in Table S2.

### 2.14 Statistical Analysis

All statistical analyses were performed using GraphPad Prism version 10.0 (GraphPad Software, San Diego, CA, USA). Data are presented as mean ± standard deviation (SD) from at least three independent experiments unless otherwise specified. For comparison between two groups, two‐tailed unpaired Student's *t*‐tests were used. For comparisons involving more than two groups, one‐way analysis of variance (ANOVA) followed by appropriate post hoc multiple comparison tests was conducted. Statistical significance was determined as *p* < 0.05.

## 3 Results

### 3.1 Standardisation of Dosage Determination

To ensure precise and reliable comparison of treatment groups, the BCA assay outcomes demonstrated that Px 1:10, Px 1:50, and Px 1:100 corresponded to 100, 20, and 10 μg/mL of exosomal total protein, respectively. Dilutions are stated in the results to facilitate clinical translation. The rhBMP2 group was treated at a physiological concentration of 100 ng/mL as previously reported in the literature [32, 33, 34, 35].

### 3.2 Px Characterisation and Cellular Uptake

**FIGURE 1:**
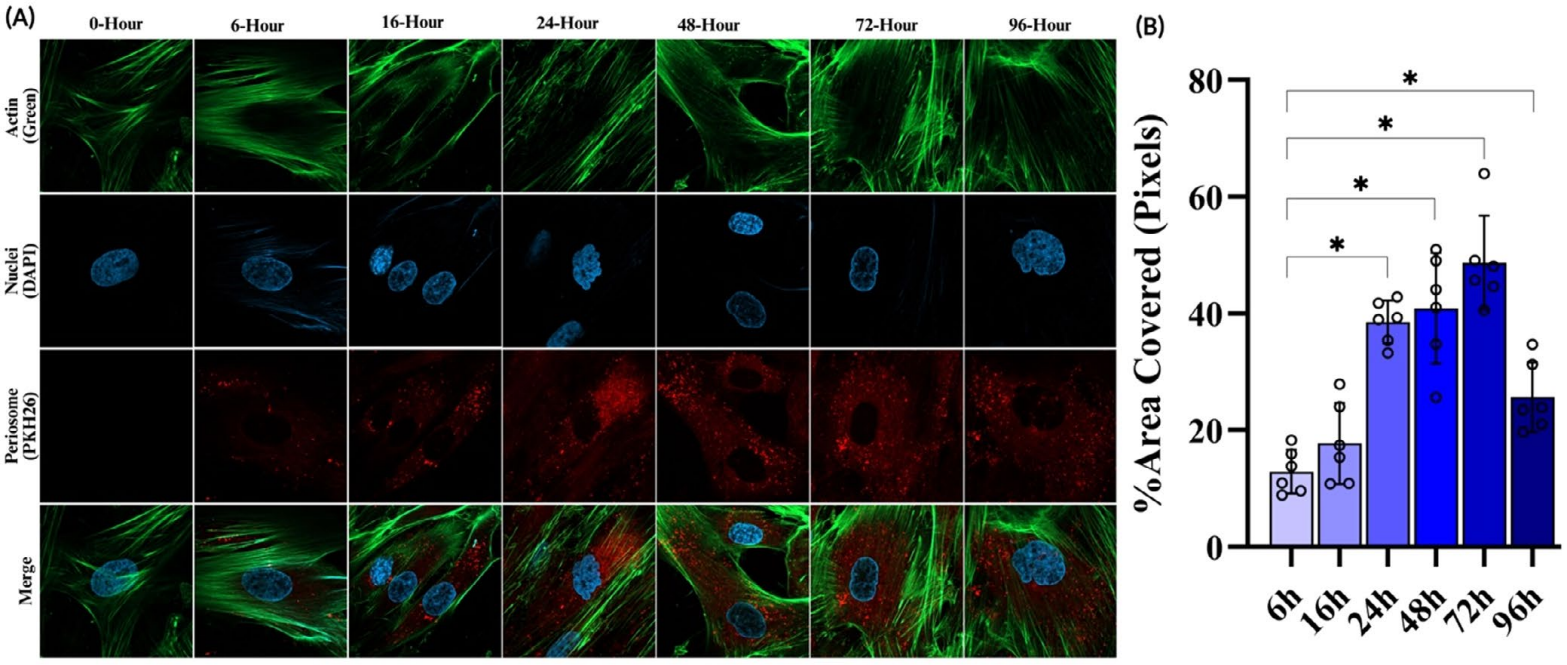
(A) Confocal microscopy was used to image immunofluorescence of the internalisation of PKH26‐labelled Px by BMSCs (63× magnification) at 0, 6, 16, 24, 48, 72, and 96 h; and (B) Semi‐quantitative analysis of cellular uptake of Px was calculated as the percent area covered by Px in Pixels. *Statistically significant difference (*p* < 0.0001).

TEM analysis revealed that Px exhibited a predominantly spheroidal to cup‐shaped morphology, with a mean diameter of ~98 nm in the isolated fractions (Figure S1). Following the incorporation of PKH67‐labelled Px into BMSC cultures, CLSM demonstrated efficient cellular uptake, with the majority of the vesicles being taken up by 72 h (Figure 1A,B). Importantly, retention of Px was also observed after 18 days of treatment, with the majority of Px residing intracellularly (Figure S2).

### 3.3 Cell Viability

**FIGURE 2:**
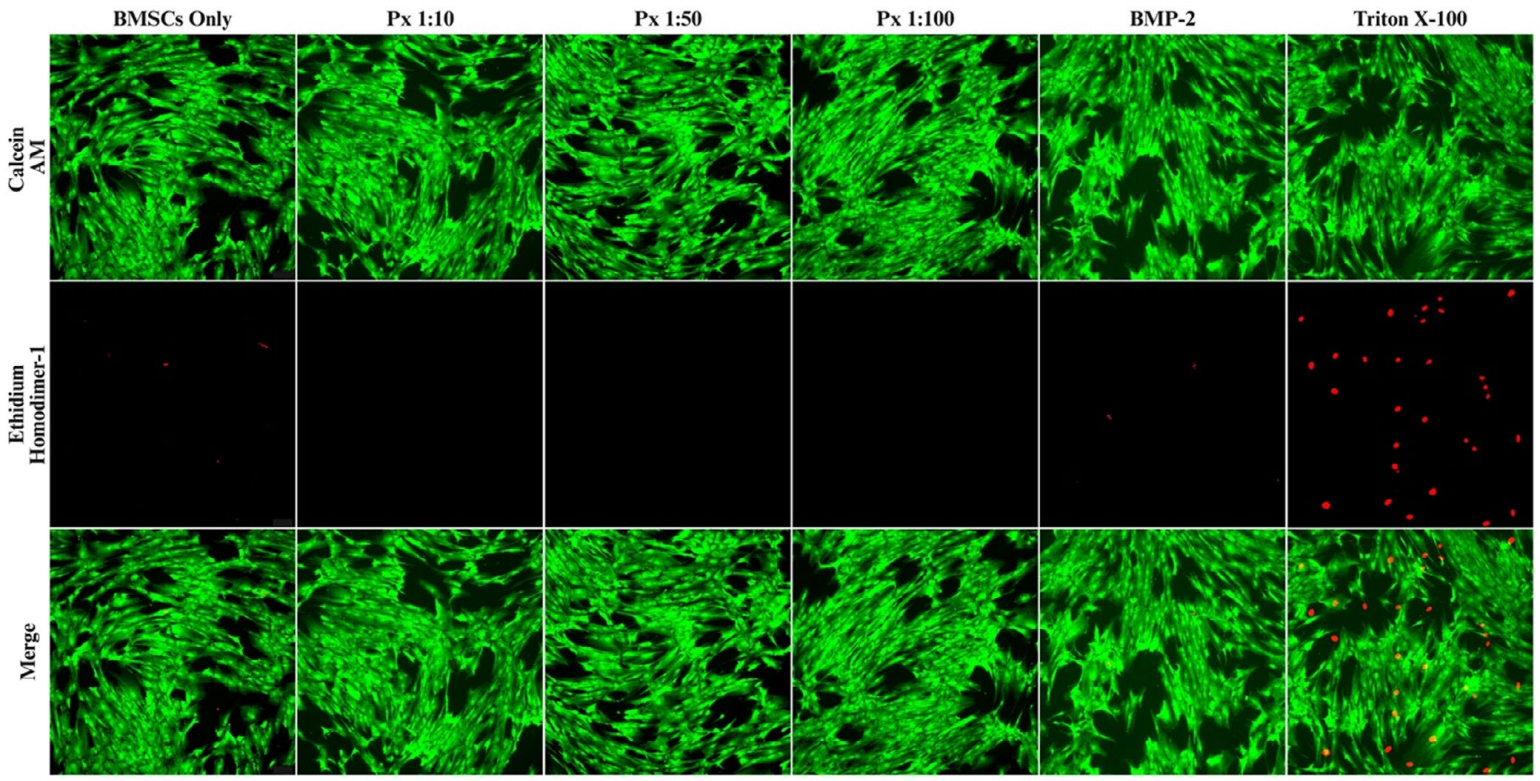
Live/dead assay's immunofluorescence images (10× magnification) of untreated BMSCs (control) and BMSCs treated with different test groups, as well as Triton X‐100.

Px‐treated BMSCs survived up to 7 days without replenishment of basal culture medium, while untreated and rhBMP2‐treated cells died in nutrient‐deprived conditions after 5 days (data not shown). After 24 h, live/dead staining showed that BMSCs in all Px‐ and rhBMP2‐treated groups retained a dense, spindle‐shaped morphology, characterised by predominant green fluorescence and minimal red signals, indicative of high cell viability compared to the control. In contrast, Triton X treatment resulted in extensive cell death (Figure 2).

### 3.4 Cell Migration Outcomes

**FIGURE 3:**
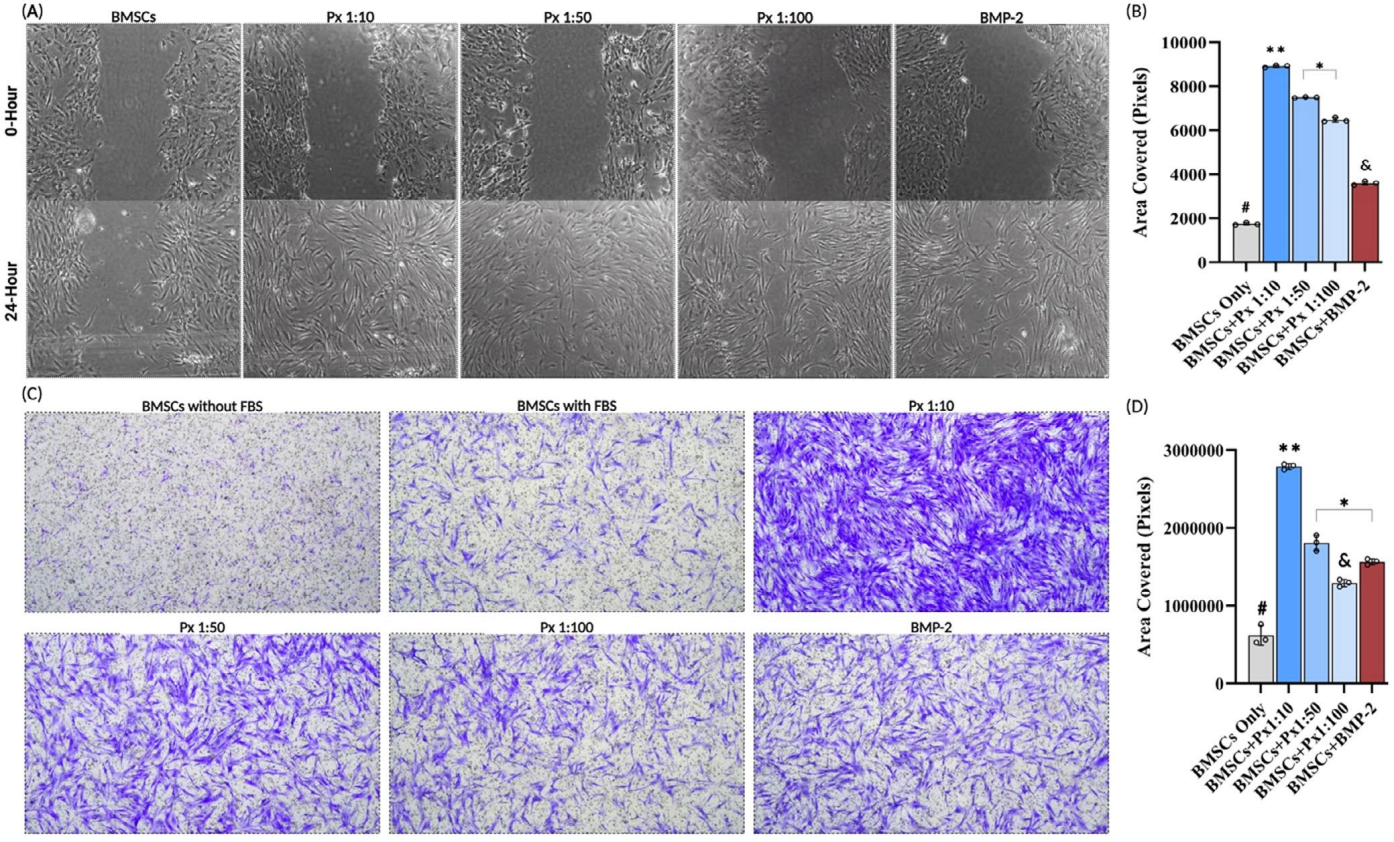
(A) Qualitative and (B) quantitative measurement of scratch wound assay (4× magnification). (C) Qualitative and (D) quantitative transwell migration assay (4× magnification) for the migratory ability of BMSCs treated by different research groups for 24 h. Semi‐quantitative analysis was performed by calculating the total area covered (Pixels) by BMSCs. ^#^Statistically significantly lower than all other groups; **statistically significantly higher than all other groups; *statistically significant difference between two groups; ^&^statistically significantly lower than all other experimental groups.

In scratch wound healing, BMSCs treated with Px showed superior migratory ability compared to rhBMP2. The extent of scratch closure following the order: Px 1:10 (8912 ± 52 pixels) > Px 1:50 (7494 ± 19 pixels) > Px 1:100 (6474 ± 102 pixels) > rhBMP2 (3595 ± 85 pixels) > control (1744 ± 59 pixels) (Figure 3A,B). Similarly, in the transwell assay, both Px‐treated groups outperformed rhBMP2 in promoting BMSC migration. Px 1:10 achieved the highest migration (353%), followed by Px 1:50 (193%), both significantly exceeding rhBMP2 (154%). Only Px 1:100 (109%) demonstrated lower migration than rhBMP2 (Figure 3C,D).

### 3.5 Alkaline Phosphatase Assay

**FIGURE 4:**
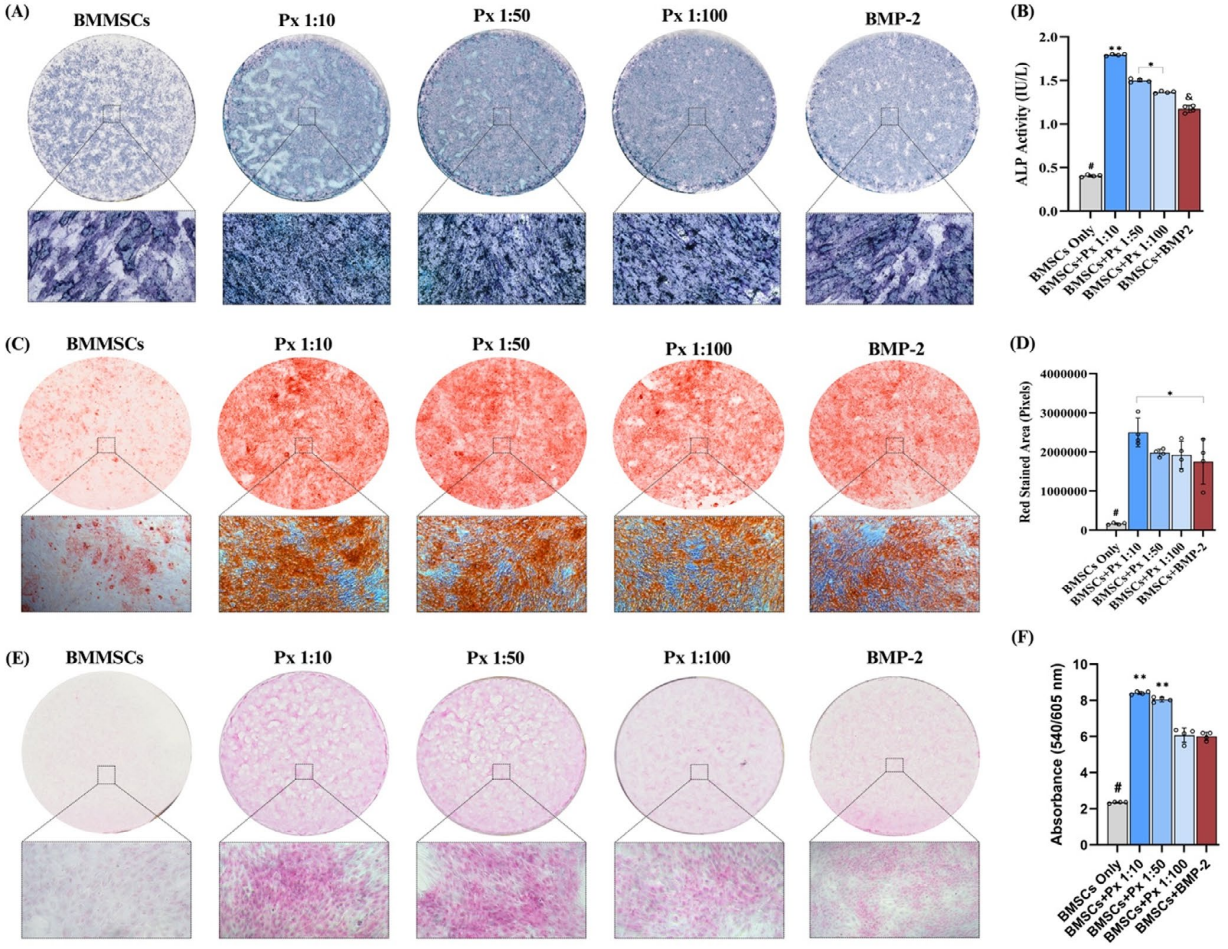
(A) Qualitative and (B) quantitative measurement of ALP activity staining of BMSCs; (C) Qualitative and (D) semi‐quantitative measurement of ARS staining of BMSCs; (E) Qualitative and (F) quantitative measurement of collagen deposition staining of BMSCs treated by different experimental groups. 1× and 10× magnification was used. ^#^Statistically significantly lower than all other groups; **statistically significantly higher than all other groups; *statistically significant difference between two groups; ^&^statistically significantly lower than all other experimental groups.

Px treatment resulted in significantly greater ALP activity compared to rhBMP2. Among Px groups, the strongest activity was found at Px 1:10 (1.85 ± 0.05 IU/L), followed by Px 1:50 (1.55 ± 0.04 IU/L) and Px 1:100 (1.40 ± 0.05 IU/L). In contrast, rhBMP2 (1.25 ± 0.06 IU/L) increased ALP staining relative to control (0.45 ± 0.04 IU/L), but its effect remained consistently lower than all Px groups (Figure 4A,B).

### 3.6 Calcium Deposition Assay

ARS staining confirmed the superior mineralization potential of Px compared to rhBMP2. Px 1:10 induced the highest calcium deposition (2.50 × 10^6^ ± 0.37 × 10^6^), with Px 1:50 (1.97 × 10^6^ ± 0.95 × 10^6^) and Px 1:100 (1.92 × 10^6^ ± 0.34 × 10^6^) also exhibiting a significant increase over control. While rhBMP2 (1.75 × 10^6^ ± 0.57 × 10^6^) enhanced mineralization compared to control (0.17 × 10^6^ ± 0.025 × 10^6^), it remained less effective than Px‐treated groups (Figure 4C,D).

### 3.7 Collagen Deposition Assay

Collagen staining further revealed the superiority of Px over rhBMP2 in extracellular matrix production. Px 1:10 (8.41 ± 0.09 μg/mL) and Px 1:50 (8.05 ± 0.13 μg/mL) achieved the greatest collagen deposition, while Px 1:100 (6.07 ± 0.40 μg/mL) and rhBMP2 (6.02 ± 0.22 μg/mL) induced comparable levels. Both treatments exceeded control (2.35 ± 0.04 μg/mL), but only Px 10 and Px 50 significantly outperformed rhBMP2 (Figure 4E,F).

### 3.8 Gene Expression

**FIGURE 5:**
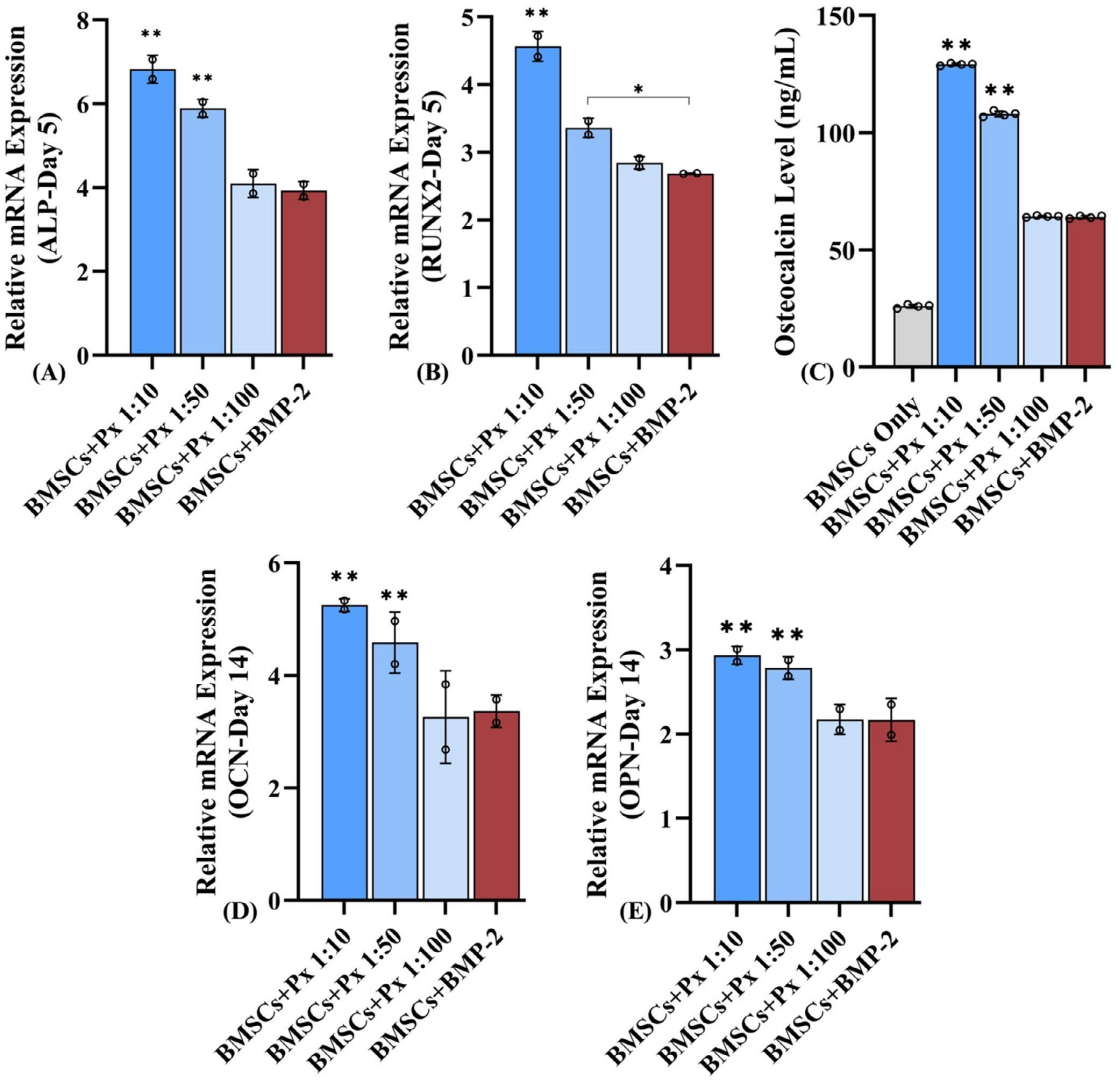
Gene expression analysis for Px 1:10, Px 1:50, Px 1:100, and rhBMP2 mediated osteogenic differentiation of BMSCs. (A) Alkaline phosphatase; (B) Runt‐related transcription factor 2; (C) Osteocalcin; and (D) Osteopontin; (E) Osteocalcin secretion ELISA assay for BMSCs treated by different research groups. **Statistically significantly higher than all other groups; *statistically significant difference between two groups.

At day 5, early osteogenic markers were upregulated in all treatment groups, but Px consistently outperformed rhBMP2. Px 1:10 induced the strongest increases (*ALP*: ~7.5‐fold; *RUNX2*: ~4.5‐fold), significantly higher than Px 1:50, Px 1:100, and rhBMP2 (*p* < 0.001). Px 1:50 and Px 1:100 demonstrated intermediate expression, while rhBMP2 induced only modest upregulation compared to Px groups (Figure 5A,B). By day 14, a similar expression trend was found for late markers. Px 1:10 produced the highest expression (*OCN*: ~5.5‐fold; *OPN*: ~3.2‐fold), surpassing Px 1:50, Px 1:100, and rhBMP2 (*p* < 0.05–0.001). Px 1:50 maintained moderate levels, while Px 1:100 and rhBMP2 showed comparatively weaker expressions (Figure 5C,D).

### 3.9 Osteocalcin Secretion

Consistent with gene expression, ELISA at day 14 confirmed greater OCN secretion in Px‐treated groups compared to rhBMP2. Px 1:10 achieved the highest secretion (~130 ng/mL; *p* < 0.0001), followed by Px 1:50 (~110 ng/mL; *p* < 0.001). Px 1:100 (~65 ng/mL) and rhBMP2 (~70 ng/mL) were comparable, both significantly lower than Px 1:10 and Px 1:50 (Figure 5E).

## 4 Discussion

Exosomes represent a novel class of bioactive exosomes with substantial translational potential in regenerative medicine and bone tissue engineering [10, 12, 14]. In contrast with rhBMP2, which requires supraphysiological doses to achieve therapeutic efficacy and is correlated with safety concerns, including rapid bioactivity loss, inflammation, and ectopic bone formation [8], exosomes offer a biologically optimised, nanoscale delivery model that can sustain osteogenic signalling [13]. As evident from the findings of this study, the delayed yet significant internalisation dynamics, combined with their sustained intracellular retention, confer an extended window of bioactivity in BMSCs, allowing consistent activation of osteogenic signalling without the requirement for repeated dosing. Furthermore, Px demonstrated a multifactorial mechanism of action, promoting cell viability, migration, mineralization, collagen formation, osteocalcin secretion, and expression of early and late osteogenic genes, hence establishing a coordinated osteogenic response that is superior to the single‐pathway activation of rhBMP2 [36].

Interestingly, most studies assessing exosome dynamics in MSCs have consistently shown that their maximal internalisation typically happens within 24 h [37, 38, 39, 40]. This rapid vesicular trafficking has been considered a hallmark of effective cell‐exosome communication in vitro [41]. Nevertheless, our results challenged this prevailing paradigm, as CLSM found a progressive and time‐dependent increase in exosome uptake, resulting in maximal intracellular accumulation at 72 h. This sustained uptake indicates that Px possess distinct compositional or structural features, potentially unique membrane protein profiles or lipid composition, that regulate their intracellular trafficking dynamics and endocytic routing in BMSCs. Such an extended temporal window of exosome uptake may prolong their functional engagements and bioavailability with target cells, hence broadening our comprehension of cell‐exosome interplay and highlighting the significance of origin‐specific exosome characteristics. Notably, this unique internalisation dynamic offers a mechanistic foundation for the sustained and superior osteogenic effects of Px in comparison with rhBMP2.

Equally important is the finding that Px persisted within BMSCs for 18 days after treatment. To the authors' knowledge, such sustained intracellular residency has not been reported for exosomes, which are usually described as transiently present in cells before undergoing exocytosis or degradation. This retention of Px over this extended time indicates a sustained and stable intracellular interplay that may consistently prime osteogenesis‐associated cascades, hence augmenting their long‐term functionality. One plausible justification is that Px may evade lysosomal degradation, either via preferential trafficking into recycling endosomes or by inherent resistance conferred by their membrane composition or other subcellular compartments, enhancing stability. This process would enable Px to serve as a reservoir for long‐term release of osteogenic signals, hence overcoming one of the primary hurdles of traditional recombinant proteins.

Another important finding was that Px‐treated BMSCs survived up to 7 days without replenishment of basal culture medium, while untreated and rhBMP2‐treated cells died in nutrient‐deprived conditions after 5 days. This extended survival highlights the cytoprotective effects of Px, which probably yield constant trophic support via the sustained release of bioactive cargos, including cytokines, regulatory RNAs, and growth factors [42]. Mechanistically, this long‐term survival may be modulated via the stimulation of pro‐survival pathways, including the inhibition of oxidative stress responses, stabilisation of mitochondrial activity, and MAPK or PI3K/AKT [43]. Contrarily, rhBMP2, while osteoinductive, was unable to confer such survival effects, underscoring a basic limitation of protein‐mediated therapies. This ability of Px to maintain BMSC viability in nutrient‐deprived conditions indicates they not only regulate osteodifferentiation but also improve cellular resistance to harsh microenvironments, a feature of vital significance for the success of regenerative approaches in vivo.

Px significantly outperformed rhBMP2 in enhancing BMSC migration. While rhBMP2 mainly regulates the osteogenic pathway, Px seems to carry a wide spectrum of bioactive signals that improve cell motility and cytoskeletal remodelling. This difference highlights the superior efficacy of Px to support early cell recruitment, a vital step in bone regeneration, in comparison with the more restricted effects of rhBMP2.

Although terminal osteogenic differentiation is usually related to decreased proliferation and migration, this association is temporally mediated and context dependent [44, 45]. During the initial phases of bone regeneration, mesenchymal progenitor cells must first experience recruitment, survival, and motility prior to committing to osteogenic lineage advancement [46, 47]. In the current report, Px concurrently improved BMSC migration and early osteogenic marker expression, suggesting that these exosomes enhance a regenerative “priming” condition instead of premature terminal differentiation. This is facilitated by the significant upregulation of ALP and RUNX2 at day 5. On the contrary, rhBMP2 predominantly stimulated differentiation without significantly promoting migratory capability [48, 49], indicating a more limited, late‐phase osteogenic signalling signature. The capability of Px to maintain migratory competence while stimulating osteogenic programming may be mechanistically associated with their prolonged intracellular residency and multifactorial signalling cargo, enabling coordinated stimulation of cytoskeletal remodelling and lineage‐targeted transcriptional cascades. This dual‐stage modulation probably underlies the superior regenerative activity of Px in comparison with rhBMP2 in this study.

Our results exhibited that Px are more efficient in enhancing osteodifferentiation of BMSCs than rhBMP2 via upregulating core markers across multiple phases of osteogenesis. Particularly, Px induced stronger early‐phase expression of *ALP* and *RUNX2*, which are vital for the propagation of osteogenesis [50, 51], alongside late‐phase expression of *OCN* and *OPN*, which mediate matrix mineralization and maturation [52, 53]. On the contrary, rhBMP2 demonstrated an effect comparable to the lowest concentration of exosomes (Px 1:100) on these markers, indicating a relatively limited ability to orchestrate both early and late osteodifferentiation.

Despite the interesting and promising findings, this study has some limitations that should be acknowledged. First, this is an in vitro report, and while Px showed superior osteo‐promotive and cytoprotective effects in comparison with rhBMP2, their behaviour in vivo within the intricate bone niche remains uninvestigated. Second, although the study demonstrated sustained retention of Px, the precise intracellular trafficking mechanisms and cascades regulating these effects were not fully investigated. Third, dose standardisation was restricted to three dilutions/concentrations, and the immunogenicity, long‐term safety, and potential off‐target impacts of Px were not evaluated. Eventually, while this study utilised rhBMP2 as a comparator at a standard concentration reported in the published literature, variations in bioavailability, delivery kinetics, and molecular stability may affect direct comparisons. Overcoming these limitations in future mechanistic and in vivo investigations will be crucial for validating the translational potential of Px for bone tissue engineering applications.

## 5 Conclusion

Px exhibited superior efficacy over rhBMP2 in promoting osteogenic differentiation of BMSCs, showing cytoprotective, pro‐migratory, and osteo‐promotive effects, highlighting them as a promising, cell‐free nanotherapeutic approach with substantial translational potential for bone tissue engineering.

## Author Contributions

P.A. contributed to study conception and design, data acquisition, analysis, and interpretation, drafted and critically revised the manuscript; D.A.S. and J.B.‐S. contributed to study design, data acquisition, and interpretation, drafted and critically revised the manuscript; N.F. contributed to study design, data analysis, and interpretation, drafted and critically revised the manuscript; N.E. contributed to study design, data analysis, and interpretation, drafted and critically revised the manuscript; R.J.M. and G.A.K. contributed to study conception, data analysis, and interpretation, drafted and critically revised the manuscript. All authors gave final approval and agreed to be accountable for all aspects of the work.

## Funding

This study was funded by Miron Research and Development in Dentistry.

## Ethics Statement

The authors have nothing to report.

## Consent

The authors have nothing to report.

## Conflicts of Interest

The authors declare no conflicts of interest.

## Supporting information


**Figure S1:** The morphology of Px under transmission electron microscopy.
**Figure S2:** Confocal microscopy was used to image immunofluorescence of the internalisation of PKH26‐labelled Px by BMSCs (63× magnification) on day 18.
**Table S1:** Key resources.
**Table S2:** List of specific primers.

## Data Availability

The data that support the findings of this study are available from the corresponding author upon reasonable request.

## References

[1] E. Della Bella , A. Buetti‐Dinh , G. Licandro , et al., “Dexamethasone Induces Changes in Osteogenic Differentiation of Human Mesenchymal Stromal Cells via SOX9 and PPARG, but Not RUNX2,” International Journal of Molecular Sciences 22 (2021): 4785, 10.3390/ijms22094785. 33946412 PMC8124248

[2] R. Agarwal and A. J. García , “Biomaterial Strategies for Engineering Implants for Enhanced Osseointegration and Bone Repair,” Advanced Drug Delivery Reviews 94 (2015): 53–62, 10.1016/j.addr.2015.03.013. 25861724 PMC4598264

[3] A. H. Schmidt , “Autologous Bone Graft: Is It Still the Gold Standard?,” Injury 52 (2021): S18–S22, 10.1016/j.injury.2021.01.043. 33563416

[4] M. Wang , Q. Yuan , and L. Xie , “Mesenchymal Stem Cell‐Based Immunomodulation: Properties and Clinical Application,” Stem Cells International 2018 (2018): 3057624, 10.1155/2018/3057624. 30013600 PMC6022321

[5] G. D. D'Ippolito , P. C. Schiller , C. Ricordi , B. A. Roos , and G. A. Howard , “Age‐Related Osteogenic Potential of Mesenchymal Stromal Stem Cells From Human Vertebral Bone Marrow,” Journal of Bone and Mineral Research 14 (1999): 1115–1122, 10.1359/jbmr.1999.14.7.1115. 10404011

[6] R. E. De La Vega , M. van Griensven , W. Zhang , et al., “Efficient Healing of Large Osseous Segmental Defects Using Optimized Chemically Modified Messenger RNA Encoding BMP‐2,” Science Advances 8 (2022): eabl6242, 10.1126/sciadv.abl6242. 35171668 PMC8849297

[7] L. Zhu , Y. Liu , A. Wang , et al., “Application of BMP in Bone Tissue Engineering,” Frontiers in Bioengineering and Biotechnology 10 (2022): 810880, 10.3389/fbioe.2022.810880. 35433652 PMC9008764

[8] A. W. James , G. LaChaud , J. Shen , et al., “A Review of the Clinical Side Effects of Bone Morphogenetic Protein‐2,” Tissue Engineering Part B: Reviews 22 (2016): 284–297, 10.1089/ten.TEB.2015.0357. 26857241 PMC4964756

[9] Y. Tang , Y. Zhou , and H.‐J. Li , “Advances in Mesenchymal Stem Cell Exosomes: A Review,” Stem Cell Research & Therapy 12 (2021): 71, 10.1186/s13287-021-02138-7. 33468232 PMC7814175

[10] P. Ahmad , N. Estrin , N. Farshidfar , Y. Zhang , and R. J. Miron , “Mechanistic Insights Into Periodontal Ligament Stem Cell‐Derived Exosomes in Tissue Regeneration,” Clinical Oral Investigations 29 (2025): 357, 10.1007/s00784-025-06422-1. 40562987 PMC12198077

[11] R. J. Miron and Y. Zhang , “Understanding Exosomes: Part 1—Characterization, Quantification and Isolation Techniques,” Periodontology 2000 94 (2024): 231–256, 10.1111/prd.12520. 37740431

[12] P. Ahmad , N. Estrin , N. Farshidfar , Y. Zhang , and R. J. Miron , “Isolation Methods of Exosomes Derived From Dental Stem Cells,” International Journal of Oral Science 17 (2025): 50, 10.1038/s41368-025-00370-y. 40523888 PMC12170887

[13] R. J. Miron , N. E. Estrin , A. Sculean , and Y. Zhang , “Understanding Exosomes: Part 2—Emerging Leaders in Regenerative Medicine,” Periodontology 2000 94 (2024): 257–414, 10.1111/prd.12561. 38591622

[14] P. Ahmad , N. Estrin , N. Farshidfar , Y. Zhang , and R. J. Miron , “Mechanistic Insights Into Dental Stem Cells‐Derived Exosomes in Regenerative Endodontics,” International Endodontic Journal 58 (2025): 1384–1407, 10.1111/iej.14269. 40497413 PMC12339802

[15] N. Estrin , N. Farshidfar , P. Ahmad , et al., “Exosome‐Mediated Alveolar Ridge Augmentation: A First Human Case Report With Histology,” International Journal of Periodontics and Restorative Dentistry 46 (2026): 30–41, 10.11607/prd.7567. 40053497

[16] R. J. Miron , N. E. Estrin , A. Sculean , and Y. Zhang , “Understanding Exosomes: Part 3—Therapeutic + Diagnostic Potential in Dentistry,” Periodontology 2000 94 (2024): 415–482, 10.1111/prd.12557. 38546137

[17] F. Diomede , S. Guarnieri , P. Lanuti , et al., “Extracellular Vesicles (EVs): A Promising Therapeutic Tool in the Heart Tissue Regeneration,” BioFactors 50 (2024): 509–522, 10.1002/biof.2025. 38131134

[18] Y. Della Rocca , F. Diomede , F. Konstantinidou , et al., “Protective Effect of Oral Stem Cells Extracellular Vesicles on Cardiomyocytes in Hypoxia‐Reperfusion,” Frontiers in Cell and Developmental Biology 11 (2024): 1260019, 10.3389/fcell.2023.1260019. 38288344 PMC10823008

[19] M. Maqsood , M. Kang , X. Wu , J. Chen , L. Teng , and L. Qiu , “Adult Mesenchymal Stem Cells and Their Exosomes: Sources, Characteristics, and Application in Regenerative Medicine,” Life Sciences 256 (2020): 118002, 10.1016/j.lfs.2020.118002. 32585248

[20] A. Hassanzadeh , H. S. Rahman , A. Markov , et al., “Mesenchymal Stem/Stromal Cell‐Derived Exosomes in Regenerative Medicine and Cancer; Overview of Development, Challenges, and Opportunities,” Stem Cell Research & Therapy 12 (2021): 297, 10.1186/s13287-021-02378-7. 34020704 PMC8138094

[21] T. Zhao , F. Sun , J. Liu , et al., “Emerging Role of Mesenchymal Stem Cell‐Derived Exosomes in Regenerative Medicine,” Current Stem Cell Research & Therapy 14 (2019): 482–494, 10.2174/1574888X14666190228103230. 30819086

[22] M. D. Hade , C. N. Suire , and Z. Suo , “Mesenchymal Stem Cell‐Derived Exosomes: Applications in Regenerative Medicine,” Cells 10 (2021): 1959, 10.3390/cells10081959. 34440728 PMC8393426

[23] H. Jing , X. He , and J. Zheng , “Exosomes and Regenerative Medicine: State of the Art and Perspectives,” Translational Research 196 (2018): 1–6, 10.1016/j.trsl.2018.01.005. 29432720

[24] Q. Yang , X. Chang , J. Y. Lee , et al., “Angle‐Controllable RNA Tiles for Programable Array Assembly and RNA Sensing,” Nature Communications 16 (2025): 3728, 10.1038/s41467-025-58938-5. PMC12009304 40253384

[25] P. Ahmad , L. M. Marin , C. Lowe , G. S. Katselis , and W. L. Siqueira , “Salivary Protein Homology Between Humans and Dogs: Mass Spectrometry‐Based Proteomics Analysis,” Journal of Dentistry 142 (2024): 104855, 10.1016/j.jdent.2024.104855. 38246308

[26] F. Lei , M. Li , T. Lin , H. Zhou , F. Wang , and X. Su , “Treatment of Inflammatory Bone Loss in Periodontitis by Stem Cell‐Derived Exosomes,” Acta Biomaterialia 141 (2022): 333–343, 10.1016/j.actbio.2021.12.035. 34979326

[27] J. Schindelin , I. Arganda‐Carreras , E. Frise , et al., “Fiji: An Open‐Source Platform for Biological‐Image Analysis,” Nature Methods 9 (2012): 676–682, 10.1038/nmeth.2019. 22743772 PMC3855844

[28] F. Bian , Y.‐W. Lan , S. Zhao , et al., “Lung Endothelial Cells Regulate Pulmonary Fibrosis Through FOXF1/R‐Ras Signaling,” Nature Communications 14 (2023): 2560, 10.1038/s41467-023-38177-2. PMC10156846 37137915

[29] S. V. Harb , N. J. Bassous , T. A. de Souza , et al., “Hydroxyapatite and β‐TCP Modified PMMA‐TiO2 and PMMA‐ZrO2 Coatings for Bioactive Corrosion Protection of Ti6Al4V Implants,” Materials Science & Engineering. C, Materials for Biological Applications 116 (2020): 111149, 10.1016/j.msec.2020.111149. 32806280

[30] J. D. Weston , B. Austin , H. Levis , et al., “CRISPRi‐Driven Osteogenesis in Adipose‐Derived Stem Cells for Bone Healing and Tissue Engineering,” bioRxiv (2022), 10.1101/2022.11.15.513563.

[31] M. S. Molla , D. R. Katti , J. Iswara , R. Venkatesan , R. Paulmurugan , and K. S. Katti , “Prostate Cancer Phenotype Influences Bone Mineralization at Metastasis: A Study Using an In Vitro Prostate Cancer Metastasis Testbed,” JBMR Plus 4 (2020): e10256, 10.1002/jbm4.10256. 32083238 PMC7017885

[32] W.‐H. Ha , H.‐S. Seong , N.‐R. Choi , B.‐S. Park , and Y.‐D. Kim , “Recombinant Human Bone Morphogenic Protein‐2 Induces the Differentiation and Mineralization of Osteoblastic Cells Under Hypoxic Conditions via Activation of Protein Kinase D and p38 Mitogen‐Activated Protein Kinase Signaling Pathways,” Tissue Engineering and Regenerative Medicine 14 (2017): 433–441, 10.1007/s13770-017-0046-1. 30603499 PMC6171616

[33] S. L. Fung , X. Wu , J. P. Maceren , Y. Mao , and J. Kohn , “In Vitro Evaluation of Recombinant Bone Morphogenetic Protein‐2 Bioactivity for Regenerative Medicine,” Tissue Engineering. Part C, Methods 25 (2019): 553–559, 10.1089/ten.TEC.2019.0156. 31418333 PMC6761583

[34] E. L. Durham , R. Kishinchand , Z. J. Grey , and J. J. Cray , “rhBMP2 Alone Does Not Induce Macrophage Polarization Towards an Increased Inflammatory Response,” Molecular Immunology 117 (2020): 94–100, 10.1016/j.molimm.2019.10.021. 31759326 PMC6931256

[35] J. Lee , J. Jang , S.‐R. Cha , et al., “Recombinant Human Bone Morphogenetic Protein‐2 Priming of Mesenchymal Stem Cells Ameliorate Acute Lung Injury by Inducing Regulatory T Cells,” Immune Network 23 (2023): e48, 10.4110/in.2023.23.e48. 38188599 PMC10767548

[36] Z. Du , S. A. Rizzo , T. L. Sarrafian , et al., “Engineered BMP2/BMP7 Extracellular Vesicles Induce Autocrine BMP Release Driving SMAD Phosphorylation to Promote Bone Formation,” NPJ Regenerative Medicine 10 (2025): 26, 10.1038/s41536-025-00405-2. 40461558 PMC12134205

[37] Q. Niu , C. Lin , S. Yang , et al., “FoxO1‐Overexpressed Small Extracellular Vesicles Derived From hPDLSCs Promote Periodontal Tissue Regeneration by Reducing Mitochondrial Dysfunction to Regulate Osteogenesis and Inflammation,” International Journal of Nanomedicine 19 (2024): 8751–8768, 10.2147/IJN.S470419. 39220194 PMC11365494

[38] J. Wang , Q. Qiao , Y. Sun , et al., “Osteogenic Differentiation Effect of Human Periodontal Ligament Stem‐Cell Initial Cell Density on Autologous Cells and Human Bone Marrow Stromal Cells,” International Journal of Molecular Sciences 24 (2023): 7133, 10.3390/ijms24087133. 37108296 PMC10138982

[39] S. Li , X. Guan , W. Yu , Z. Zhao , Y. Sun , and Y. Bai , “Effect of Human Periodontal Ligament Stem Cell‐Derived Exosomes on Cementoblast Activity,” Oral Diseases 30 (2024): 2511–2522, 10.1111/odi.14671. 37448205

[40] Y. Shi , R. Zhang , N. Da , et al., “Aspirin Loaded Extracellular Vesicles Inhibit Inflammation of Macrophages via Switching Metabolic Phenotype in Periodontitis,” Biochemical and Biophysical Research Communications 667 (2023): 25–33, 10.1016/j.bbrc.2023.05.024. 37207561

[41] K. M. Hirosawa , Y. Sato , R. S. Kasai , et al., “Uptake of Small Extracellular Vesicles by Recipient Cells Is Facilitated by Paracrine Adhesion Signaling,” Nature Communications 16 (2025): 2419, 10.1038/s41467-025-57617-9. PMC11903687 40075063

[42] R. A. Kore , J. C. Henson , R. N. Hamzah , et al., “Molecular Events in MSC Exosome Mediated Cytoprotection in Cardiomyocytes,” Scientific Reports 9 (2019): 19276, 10.1038/s41598-019-55694-7. 31848380 PMC6917778

[43] S. Keshtkar , M. Kaviani , F. S. Sarvestani , et al., “Exosomes Derived From Human Mesenchymal Stem Cells Preserve Mouse Islet Survival and Insulin Secretion Function,” EXCLI Journal 19 (2020): 1064–1080, 10.17179/excli2020-2451. 33013264 PMC7527509

[44] M. Galindo , J. Pratap , D. W. Young , et al., “The Bone‐Specific Expression of Runx2 Oscillates During the Cell Cycle to Support a G1‐Related Antiproliferative Function in Osteoblasts,” Journal of Biological Chemistry 280 (2005): 20274–20285, 10.1074/jbc.M413665200. 15781466 PMC2895256

[45] D. M. Thomas , S. A. Johnson , N. A. Sims , et al., “Terminal Osteoblast Differentiation, Mediated by runx2 and p27 KIP1, Is Disrupted in Osteosarcoma,” Journal of Cell Biology 167 (2004): 925–934, 10.1083/jcb.200409187. 15583032 PMC2172443

[46] A. Sardar , S. Verma , A. Raj , B. Maji , and R. Trivedi , “Comparison of Differences in Cell Migration During the Osteogenic and Adipogenic Differentiation of the Bone Marrow‐Derived Stem Cells,” Journal of Bone Metabolism 32 (2025): 69–82, 10.11005/jbm.25.841. 40537102 PMC12183369

[47] T. Fujita , Y. Azuma , R. Fukuyama , et al., “Runx2 Induces Osteoblast and Chondrocyte Differentiation and Enhances Their Migration by Coupling With PI3K‐Akt Signaling,” Journal of Cell Biology 166 (2004): 85–95, 10.1083/jcb.200401138. 15226309 PMC2172136

[48] A. Yamaguchi , K. Sakamoto , T. Minamizato , K. Katsube , and S. Nakanishi , “Regulation of Osteoblast Differentiation Mediated by BMP, Notch, and CCN3/NOV,” Japanese Dental Science Review 44 (2008): 48–56, 10.1016/j.jdsr.2007.11.003.

[49] S. Liu , Y. Liu , L. Jiang , et al., “Recombinant Human BMP‐2 Accelerates the Migration of Bone Marrow Mesenchymal Stem Cells via the CDC42/PAK1/LIMK1 Pathway In Vitro and In Vivo,” Biomaterials Science 7 (2019): 362–372, 10.1039/c8bm00846a. 30484785

[50] C. M. Darjanki , C. Prahasanti , B. Kusumawardani , I. K. Wijaksana , and M. Aljunaid , “RUNX2 and ALP Expression in Osteoblast Cells Exposed by PMMA‐HAp Combination: An In Vitro Study,” Journal of Oral Biology and Craniofacial Research 13 (2023): 277–282, 10.1016/j.jobcr.2023.02.007. 36896352 PMC9988561

[51] T. M. Liu and E. H. Lee , “Transcriptional Regulatory Cascades in Runx2‐Dependent Bone Development,” Tissue Engineering. Part B, Reviews 19 (2013): 254–263, 10.1089/ten.TEB.2012.0527. 23150948 PMC3627420

[52] G. R. Beck, Jr. , B. Zerler , and E. Moran , “Phosphate Is a Specific Signal for Induction of Osteopontin Gene Expression,” Proceedings of the National Academy of Sciences of the United States of America 97 (2000): 8352–8357, 10.1073/pnas.140021997. 10890885 PMC26951

[53] S. Bailey , G. Karsenty , C. Gundberg , and D. Vashishth , “Osteocalcin and Osteopontin Influence Bone Morphology and Mechanical Properties,” Annals of the New York Academy of Sciences 1409 (2017): 79–84, 10.1111/nyas.13470. 29044594 PMC5730490

