# Supplementary material for: Effects of Periodontal‐Specific Exosomes and rhBMP2 on Osteogenic Behaviour and Differentiation of BMSCs
Source: J Cell Mol Med. 2026 Jan 28;30(3):e71039. doi: 10.1111/jcmm.71039 (PMC12852057; doi:10.1111/jcmm.71039)
Supplement: Supplementary file 1 — Figure S1: The morphology of Px under transmission electron microscopy. Figure S2: Confocal microscopy was used to image immunofluorescence of the internalisation of PKH26‐labelled Px by BMSCs (63× magnification) on day 18. Table S1: Key resources. Table S2: List of specific primers. [file JCMM-30-e71039-s001.docx]

**Supplementary Materials**

**Effects of Periodontal-Specific Exosomes in Comparison to rhBMP2 on osteogenic behavior and differentiation of BMSCs In-Vitro**

Paras Ahmad^1,2,3*^, Danyal A. Siddiqui^1,2^, Jared Bianchi-Smak^4^, Nima Farshidfar^5^, Nathan Estrin^6^, Richard J. Miron^5*^, Georgios A. Kotsakis^1,2^

^1^Department of Oral Biology, Rutgers School of Dental Medicine, The State University of New Jersey, Newark, NJ, USA;

^2^Clinical Research Center, Rutgers School of Dental Medicine, The State University of New Jersey, Newark, NJ, USA;

^3^Department of Research, Advanced PRF Education, Jupiter, FL, USA;

^4^Department of Biological Sciences, Rutgers University, Newark, NJ, USA;

^5^Department of Periodontology, University of Bern, Bern, Switzerland;

^6^Lake Erie College of Osteopathic Medicine School of Dental Medicine, Bradenton, FL, USA.

**Supplementary Figures**

**
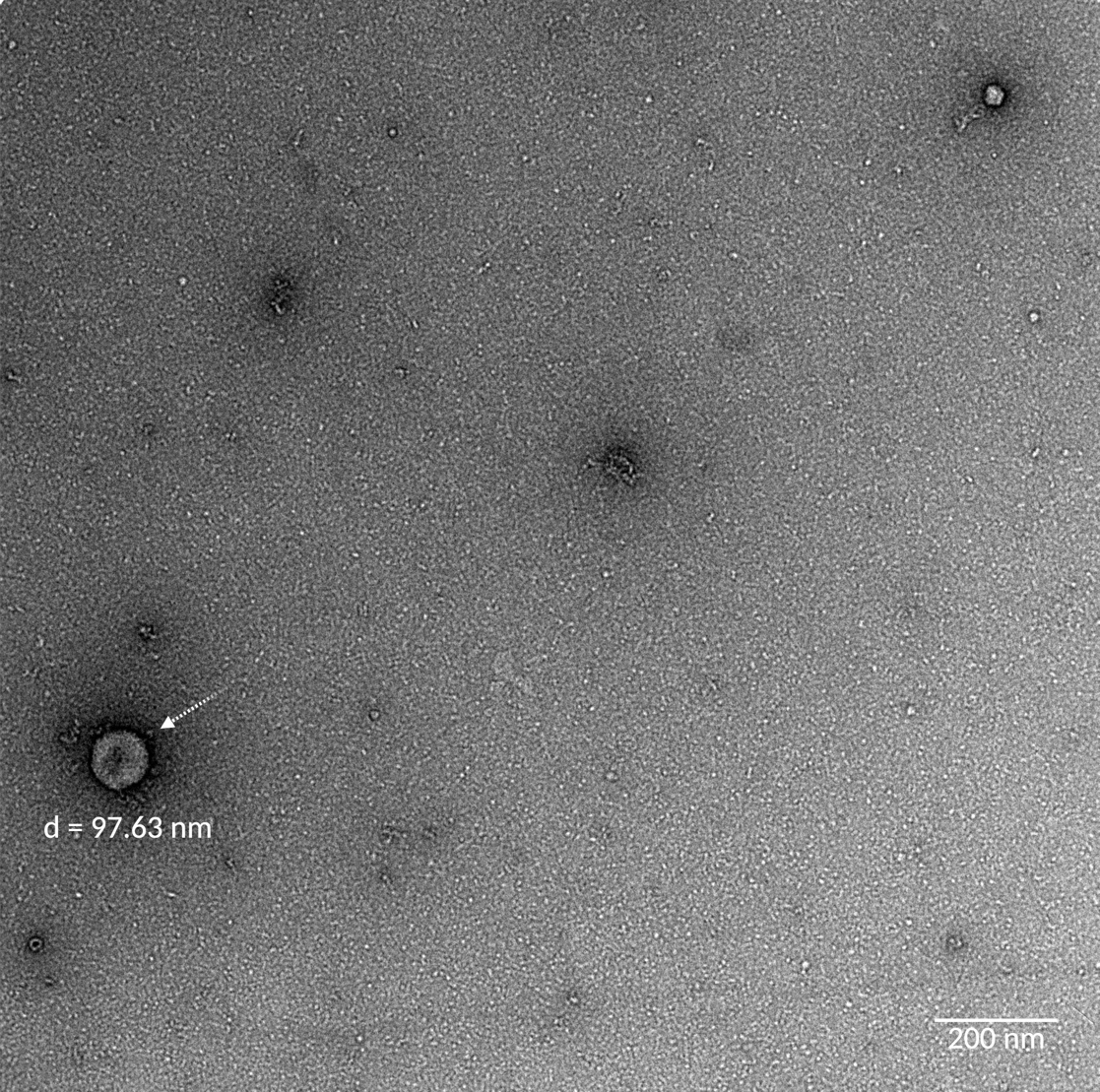
**

**Figure S1**. The morphology of Px under transmission electron microscopy.

**
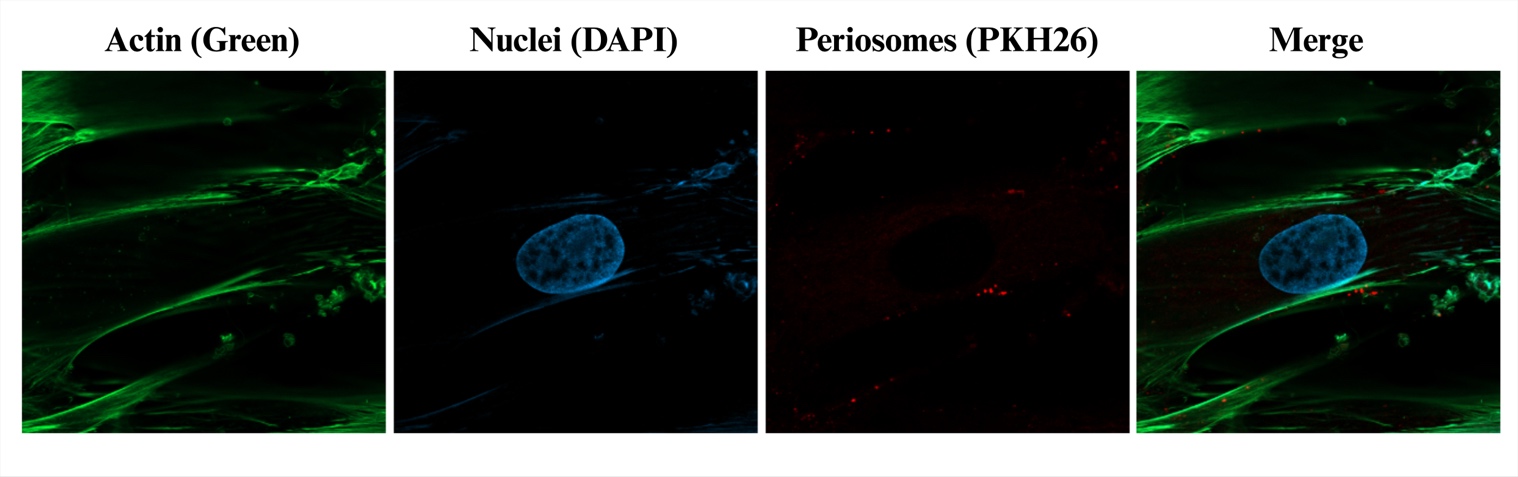
**

**Figure S2.** Confocal microscopy was used to image immunofluorescence of the internalization of PKH26-labeled Px by BMSCs (63× magnification) on day 18.

**Supplementary Tables**

**Table S1**. Key resources.

| **Reagent or Resource** | **Source** | **Identifier** |
| --- | --- | --- |
| Bone marrow mesenchymal stem cells | MilliporeSigma | SCC034 |
| Mesenchymal stem cell expansion medium | MilliporeSigma | SCM015 |
| Trypsin-EDTA solution (0.25% w/v) | Thermo Fisher Scientific | 25200-056 |
| Periosomes | Neobiosis LLC | N/A |
| Pierce^™^ BCA protein assay kit | Thermo Fisher Scientific | 23225 |
| Bovine serum albumin | Sigma-Aldrich | A8412 |
| PKH26 red fluorescent cell linker midi kit | Sigma-Aldrich | MIDI26 |
| Phosphate-buffered saline | Thermo Fisher Scientific | 10010-023 |
| Paraformaldehyde (37%) | Sigma-Aldrich | 252549 |
| ActinGreen^™^ 488 ReadyProbes^™^ reagent | Thermo Fisher Scientific | R37110 |
| NucBlue^™^ Fixed Cell Stain ReadyProbes^™^ reagent | Thermo Fisher Scientific | R37606 |
| Calcein-AM | Sigma-Aldrich | 206700 |
| Propidium iodide | Sigma-Aldrich | P4864 |
| Recombinant human bone morphogenetic protein-2 | R & D Systems | 355-BM |
| Cell counting kit-8 | Abcam | ab228554 |
| Transwell inserts with 8-μm pore filters | Avantor, VWR | 10769-212 |
| Crystal violet | Sigma-Aldrich | C6158-50G |
| StemPro^®^ osteogenesis differentiation kit | Thermo Fisher Scientific | A1007201 |
| Sirius red/fast green collagen staining kit | Chondrex Inc. | 9046 |
| ALP staining kit | Abcam | ab242287 |
| ALP colorimetric assay kit | Sigma-Aldrich | MAK447 |
| Triton^™^ X-100 | EMD Millipore | 648463 |
| Alizarin red S | Sigma-Aldrich | TMS-008 |
| Osteocalcin ELISA kit | Thermo Fisher Scientific | KAQ1381 |
| RNA extraction kit | Zymo Research | R1054 |
| RNA quantification kit | Thermo Fisher Scientific | Q10210 |
| cDNA synthesis kit | BioRad | 1708891 |

**Table S2**. List of specific primers.

| **Gene** | **Sequence** |
| --- | --- |
| *GADPH* | F: 5’ GTCTCCTCTGACTTCAACAGCG 3’  R: 5’ ACCACCCTGTTGCTGTAGCCAA 3’ |
| *ALP* | F: 5’ CCTGATGGAGATGACAGAGGCT 3’  R: 5’ TCAGTGAGTGCCTGGTAAGCCA 3’ |
| *RUNX2* | F: 5’ CCCAGTATGAGAGTAGGTGTCC 3’  R: 5’ GGGTAAGACTGGTCATAGGACC 3’ |
| *OCN* | F: 5’ CGCTACCTGTATCAATGGCTGG 3’  R: 5’ CTCCTGAAAGCCGATGTGGTCA 3’ |
| *OPN* | F: 5’ CGAGGTGATAGTGTGGTTTATGG 3’  R: 5’ GCACCATTCAACTCCTCGCTTTC 3’ |
